# 

**Published:** 1891-10

**Authors:** 


### CONTENTS FOR OCTOBER, 1891.

ORIGINAL COMMUNICATIONS.

Affections of Sight from Injuries. By ALVIN A. HUBBELL, M. D. .... 129
Rhus Aromatica in the Treatment of Incontinence of Urine. By WILLIAM C. KRAUSS, M. D. 137
Three Cases of Electrical Traumatic Neurosis. By JAMES W. PUTNAM, M. D. .... 140

CLINICAL REPORT.

A Case of Acute General Peritonitis, Treated by Morphine in Large Doses.—Recovery...... 142

SOCIETY PROCEEDINGS.

Philadelphia County Medical Society .... 144
Buffalo Medical and Surgical Association .... 156

PROGRESS IN MEDICAL SCIENCE.

Surgery .... 159

NEW INSTRUMENTS.

New Ear Scissors .... 163

EDITORIAL.

The Late Dr. Frank H. Potter .... 164
The Sheppard Hospital for the Insane .... 177
State Medical Examining and Licensing Board .... 179
The American Association of Obstetricians and Gynecologists .... 181
The Opium Treatment of Peritonitis .... 182
Medical Practice in Connecticut .... 182
The Exempts .... 183
A Medical Mecca .... 184

PERSONAL.

Dr. William C. Bailey—Dr. Julius Pohlman—Dr. John B. Hamilton—Dr. A. A. Hubbell.... 184

SOCIETY MEETINGS.

The District Medical Societies of Northern Ohio .... 185
Congress of American Physicians and Surgeons .... 185
New York State Society of Railway Surgeons .... 185

JOURNALISTIC NOTES.

Dr. Bransford Lewis at Home .... 186

BOOK REVIEWS.

Principles of Surgery .... 187
Wood's Medical and Surgical Monographs .... 188
Annual of the Universal Medical Sciences .... 188
Manual of Clinical Diagnosis .... 189
Books and Pamphlets Received .... 190

